# The view from the trenches

**DOI:** 10.1186/s12954-015-0078-6

**Published:** 2015-10-16

**Authors:** Jimmy Dorabjee

**Affiliations:** The Asian Network of People who Use Drugs (ANPUD), Bangkok, Thailand

## Commentary

Almost 25 years have passed since the emergence of Harm Reduction in the Asian region. The first harm reduction programmes began as social movements that were driven by NGOs and civil society concerned about the potential spread of HIV epidemics among people who inject drugs (PWID).

More than half the global population lives in the Asian region, with two of the most densely populated countries in the world, China and India, contributing a large proportion of the total. The two largest illicit opium-producing regions of the world, the Golden Triangle and Golden Crescent, are located in South and Southeast Asia, ensuring the abundant and widespread availability of opium and its alkaloids morphine and heroin. Until a decade ago, India was the world’s largest producer of licit opium for medicinal purposes and a proportion of opium is diverted to the domestic market for illicit opiates.

Until the mid-1970s, cannabis and opium were the two most common drugs that were traditionally used across Asia, predominantly through oral ingestion. However, aggressive marketing and widespread availability of good-quality heroin in the early and mid-1980s saw a surge in the use of oral and injected heroin while traditional opium use declined. In South Asia, in addition to the smoking and injecting of heroin, a trend of injecting pharmaceutical products such as buprenorphine and diazepam began. Over the past 15 years, South East Asia witnessed an explosive increase in the production and use of amphetamine-type stimulants (ATS) especially among the younger population, nightclub workers, sex workers, comfort workers, seafarers, truck drivers and labourers and ATS use continues to rise.

Except for a handful of small-scale NGO-run programmes in a few countries in the 1990s, harm reduction emerged late in the Asian region as a response to the threat of widespread HIV infection among people who inject drugs and from them to their sexual partners and the general community. Further, harm reduction became acceptable to Asian governments only after a series of explosive HIV epidemics among PWID had already been well established and continued to spread to new populations in several countries. Despite a growing acceptance of the effectiveness of harm reduction as a public health approach to the prevention and control of HIV, the scale and coverage of harm reduction programmes has been unable to match the rapid spread of HIV and hepatitis C infection in PWID.

In 1994, I was among a group of Asians invited to participate in the first harm reduction workshop in Kathmandu organised and facilitated by Dr. Nick Crofts, Dr. Alex Wodak and the late Aaron Peak. The workshop was an eye-opener for many of us and gave a form and name to the interventions we were implementing.

While much has been written on harm reduction, there has not been a publication focused solely on harm reduction in Asia. The Special Edition of the *Harm Reduction Journal* is a breath of fresh air and a very welcome initiative. The compilation of papers that lie within the covers takes us on a harm reduction journey from Iran and Afghanistan to China and Vietnam. The contributors are well known, respected and credible authors who many of us will recognise. I congratulate the editors and authors for bringing focus to Harm Reduction in Asia, and I am confident the special issue will be of great interest to the reader.

I would like to leave you with some issues of great concern to people who use drugs in Asia who live each day oppressed by stigma and discrimination, compulsory treatment centres, death penalty for drug offences, police brutality, poor access and availability of harm reduction services, HIV and hepatitis C infection and human rights violations. How much longer must we wait to be free from them?

